# Alternative Splicing Enhances the Transcriptome Complexity of *Liriodendron chinense*

**DOI:** 10.3389/fpls.2020.578100

**Published:** 2020-09-23

**Authors:** Zhonghua Tu, Yufang Shen, Shaoying Wen, Yaxian Zong, Huogen Li

**Affiliations:** ^1^ Key Laboratory of Forest Genetics & Biotechnology of Ministry of Education, Nanjing Forestry University, Nanjing, China; ^2^ Co-Innovation Center for Sustainable Forestry in Southern China, Nanjing Forestry University, Nanjing, China

**Keywords:** *L. chinense*, hybrid sequencing, alternative splicing, long noncoding RNAs, serine/arginine-rich genes, heterogeneous nuclear ribonucleoprotein genes, premature termination codon, weighted gene coexpression network analysis

## Abstract

Alternative splicing (AS) plays pivotal roles in regulating plant growth and development, flowering, biological rhythms, signal transduction, and stress responses. However, no studies on AS have been performed in *Liriodendron chinense*, a deciduous tree species that has high economic and ecological value. In this study, we used multiple tools and algorithms to analyze transcriptome data derived from seven tissues *via* hybrid sequencing. Although only 17.56% (8,503/48,408) of genes in *L. chinense* were alternatively spliced, these AS genes occurred in 37,844 AS events. Among these events, intron retention was the most frequent AS event, producing 1,656 PTC-containing and 3,310 non-PTC-containing transcripts. Moreover, 183 long noncoding RNAs (lncRNAs) also underwent AS events. Furthermore, weighted gene coexpression network analysis (WGCNA) revealed that there were great differences in the activities of transcription and post-transcriptional regulation between pistils and leaves, and AS had an impact on many physiological and biochemical processes in *L. chinense*, such as photosynthesis, sphingolipid metabolism, fatty acid biosynthesis and metabolism. Moreover, our analysis showed that the features of genes may affect AS, as AS genes and non-AS genes had differences in the exon/intron length, transcript length, and number of exons/introns. In addition, the structure of AS genes may impact the frequencies and types of AS because AS genes with more exons or introns tended to exhibit more AS events, and shorter introns tended to be retained, whereas shorter exons tended to be skipped. Furthermore, eight AS genes were verified, and the results were consistent with our analysis. Overall, this study reveals that AS and gene interaction are mutual—on one hand, AS can affect gene expression and translation, while on the other hand, the structural characteristics of the gene can also affect AS. This work is the first to comprehensively report on AS in *L. chinense*, and it can provide a reference for further research on AS in *L. chinense*.

## Introduction

Alternative splicing (AS) is an important post-transcriptional regulation mechanism that can result in the production of multiple mature mRNAs from a single gene, and it substantially increases the plasticity of the transcriptome and the diversity of the proteome (Marquez et al., 2012; Vitulo et al., 2014). Moreover, AS can influence the stability of mRNA by introducing a premature termination codon (PTC) into the coding sequence; transcripts with PTCs are degraded through the nonsense-mediated decay (NMD) pathway (Wang et al., 2014; Szakonyi and Duque, 2018; Chaudhary et al., 2019a). Furthermore, AS plays roles in regulating gene expression by changing the efficiency of transcription elongation and/or translation (Laloum et al., 2018; Szakonyi and Duque, 2018). Although the functions of alternative splicing in animals and plants are similar, some differences exist between them in some aspects. For example, intron retention (IR) has been reported to be the most frequent AS event in plants, whereas exon skipping (ES) is the major type in humans and animals (Xu et al., 2002; Wang et al., 2008; Ruan et al., 2018; Ma et al., 2019; Wang Z. et al., 2019). The *Suppressor of Morphological defects on Genitalia* (*SMG*) gene family (*SMG 1–7*), which plays an important role in the AS-coupled-NMD pathway, has been widely reported in animals; however, no *SMG 5* and *SMG 6* paralogs have been identified in plants. Even in plants, *SMG 1* does not appear in *Arabidopsis thaliana* but rather in *Physcomitrella patens* (Riehs et al., 2008; Lloyd and Davies, 2013; Capitao et al., 2018). These studies indicate that AS has both universality and species diversity.

In plants, AS is a universal phenomenon with pivotal roles in regulating plant growth and development, flowering, biological rhythms, signal transduction, and stress responses (Filichkin et al., 2015; Sun and Xiao, 2015; Gallegos, 2018; Ruan et al., 2018; Szakonyi and Duque, 2018; Wang et al., 2018; Calixto et al., 2019; Park et al., 2019; Yang et al., 2019). For example, in peanut (*Arachis hypogaea*), *Arabidopsis thaliana*, soybean (*Glycine max*), tomato (*Solanum lycopersicum*) and moso bamboo (*Phyllostachys edulis*), approximately 37%, 61%, 63%, 65%, and 49% of multi-exon genes undergo AS events, respectively (Marquez et al., 2012; Shen et al., 2014; Ruan et al., 2018; Zhao et al., 2018; Clark et al., 2019). Furthermore, researchers have found that AS also plays important roles in male and female gametogenesis, seed germination in *A. thaliana*, plant-pathogen interactions in wheat (*Triticum aestivum*), and mineral nutrient homeostasis maintenance in rice (*Oryza sativa*) (Dong et al., 2018; Forsthoefel et al., 2018; Tognacca et al., 2019; Zhang H. et al., 2019). These findings indicate that AS is essential for plant survival and reproduction. However, no systematic studies on AS in *L. chinense* have been conducted.

Splicing factors (SFs), such as serine/arginine-rich (SR) proteins and heterogeneous nuclear ribonucleoprotein (hnRNP), play pivotal roles in regulating AS (Chen and Manley, 2009). SR proteins are RNA-binding proteins containing one or two motifs (RNA-recognition motif) and one C-terminal domain (arginine- and serine-rich domain) (Mai et al., 2016). hnRNPs usually contain RNA-binding domains at the N-terminal and a glycine-rich domain at the C-terminal (Talukdar et al., 2011). SR proteins regulate AS by participating in spliceosome formation, whereas hnRNPs function as activators or repressors (Mauger et al., 2008; Mai et al., 2016). However, the influence of SR proteins and hnRNPs on AS genes in *L. chinense* has not been illustrated.

Long noncoding RNAs (lncRNAs) are RNA molecules with lengths greater than 200 nt that lack the ability to encode proteins (Liu et al., 2018). In plants, lncRNAs are critical regulators of growth and development, the stress response, flowering, and other biological processes (Karlik and Gozukirmizi, 2018; Liu et al., 2018; Ayachit et al., 2019; Wang P. et al., 2019). For instance, researchers have found that *lncWOX5* and *lncWOX11* participate in the regulation of poplar root development, and *lncWOX5* negatively regulates *WOX5* while *lncWOX11* positively regulates *WOX11* (Liu et al., 2018). In barley (*Hordeum vulgare*), the lncRNA *AK370841* participates in the vitamin B6 salvage pathway (Karlik and Gozukirmizi, 2018). Moreover, lncRNAs can undergo AS. Researchers have found that during rice seed development, AS events occur in lncRNAs with complex gene structures, and exon retention (as opposed to ES) is enhanced in embryos (Kiegle et al., 2018). A study on *A. thaliana* revealed that AS of some lncRNAs is closely related to small temperature changes (Calixto et al., 2019). However, research on AS of lncRNAs in *Liriodendron chinense* has not been reported, and this knowledge gap needs to be bridged.

With the development of sequencing technologies, an increasing number of studies have been conducted on the transcriptomes of different species. Such species include lotus (*Nelumbo nucifera*), tomato, peanut, maize (*Zea mays*), moso bamboo, *Passiflora edulis* Sims, and *Cinnamomum camphora* (Liu et al., 2017; Min, 2017; Chen C. et al., 2018; Ruan et al., 2018; Wang et al., 2018; Zhao et al., 2018; Clark et al., 2019; Zhang Y. et al., 2019). Moreover, using weighted gene coexpression network analysis (WGCNA) to reveal the relationship between genes based on sequencing data has become very popular. WGCNA is a systematic biology method applied in constructing gene coexpression networks which can be further divided into different coexpression modules (Zhang and Horvath, 2005; Langfelder and Horvath, 2008). Genes from the same coexpression module usually have similar expression patterns and closely related functions (Jia et al., 2020). Researchers can associate coexpression modules with different traits to reveal the relationships between gene sets and phenotypes, and this method had been widely applied in the fields of medicine and plant science (Wang J.-L. et al., 2019; Zhu et al., 2019; Esmaeili et al., 2020; Jia et al., 2020). However, using WGCNA to reveal the AS gene coexpression module in *L. chinense* had not been reported.

*L. chinense*, a deciduous tree species with beautiful flowers is widely distributed in northern Vietnam and southern China (Hao et al., 1995). Due to its high economic and ecological value, many studies have been conducted on *L. chinense* (Xu et al., 2017; Tu et al., 2019). Transcriptome analyses of *L. chinense* have also been performed (Yang et al., 2014; Workman et al., 2018). Importantly, sequencing of the *L. chinense* genome was completed in 2018 (Chen J. et al., 2018). Although researchers have obtained large amounts of transcriptome data and genome data, none have studied AS in *L. chinense*. The numbers of genes and lncRNAs that undergo AS, the frequencies of different AS events, and potential differences in AS between different tissues in *L. chinense* remain unknown.

To further investigate these topics, we used Illumina sequencing combined with single-molecule long-read sequencing technology (single-molecule real-time [SMRT] sequencing) to obtain transcriptome data for seven *L. chinense* tissues. After processing the data with multiple software programs and tools, we investigated the AS events that occurred in genes and lncRNAs, identified the differences between AS genes and non-AS genes in *L. chinense*, and determined the factors that may have caused these differences. Notably, this is the first study on AS in *L. chinense*. Our findings provide new insights into the transcriptome complexity of *L. chinense* and will serve as a reference for subsequent studies of AS in *L. chinense*.

## Materials and Methods

### Plant Materials and RNA Isolation

An adult *L. chinense* tree (26 years old) from a provenance trial plantation in Xiashu, Jurong County, Jiangsu Province (119°13′E, 32°7′N), Lushan Mountain, was used as a source tree, and Jiangxi Province (116°0′E, 29°32′N) was the provenance of the source tree. We collected seven *L. chinense* tissue type: leaves, shoot apices, sepals, bracts, petals, pistils and stamens. Each type of sample had three replicates. All 21 samples were stored in a −80°C freezer after being flash-frozen in liquid nitrogen. An RNAprep Pure Plant Kit (Tiangen, Beijing, China) was used to isolate total RNA under the direction of the product manual. Then, we used 1% agarose gels to detect the degradation and contamination of RNA, and RNA quality was detected on a NanoPhotometer^®^ spectrophotometer (IMPLEN, CA, USA). The Qubit^®^2.0 Fluorometer (Life Technologies, CA, USA) was applied to measure the RNA concentration, and the Agilent Bioanalyzer 2100 system (Agilent Technologies, CA, USA) was applied to evaluate the integrity of RNA. Only RNA samples with high quality and integrity (integrity number > 0.75) were used for library construction.

### Construction of Libraries and Transcriptome Sequencing

For SMRT sequencing, total RNA from 21 samples was mixed equally. Then, a SMARTer™ PCR cDNA Synthesis Kit (Clontech, CA, USA) was used to synthesize cDNA from three micrograms of total RNA. Subsequently, we performed polymerase chain reaction (PCR) amplification, followed by fragment selection to classify the fragments into two categories, ≤ 4 kb and > 4 kb, by using the BluePippin Size Selection System (Sage Science, MA, USA). After damage repair, end repair, linker ligation, exonuclease digestion, primer binding, and DNA polymerase binding, we constructed two SMRT bell libraries. When the construction of SMRT libraries were completed, a Qubit^®^2.0 Fluorometer (Life Technologies, CA, USA) and an Agilent Bioanalyzer 2100 system (Agilent Technologies, CA, USA) were used to quantify and measure the libraries, respectively. Then, the two libraries were used to perform SMRT sequencing on a PacBio Sequel platform (Pacific Bioscience, CA, USA).

For Illumina sequencing, a NEBNext^®^ Ultra™ RNA Library Prep Kit (NEB, Frankfurt, Germany) was used to construct libraries from three micrograms of total RNA. Briefly, we used magnetic beads with Oligo (dT) to enrich mRNA. After that, fragmentation buffer was used to break the mRNA into short fragments. Then, these mRNA fragments were used as a template with random hexamers to synthesize first-strand cDNA. After adding buffer, dNTPs, and DNA polymerase I, the second-strand cDNA was synthesized, and the AMPure XP system (Beckman Coulter, CA, USA) was used to purify double-stranded cDNA. After undergoing terminal repair, NEBNext Adaptor ligation, and size selection, the cDNA fragments were used as input materials for PCR amplification followed by PCR product purification. When the construction of the library was completed, the Bioanalyzer 2100 system (Agilent Technologies, CA, USA) was used to evaluate the quality of the library. We constructed a total of 21 Illumina libraries. Then, Illumina sequencing was performed on the Illumina HiSeq 2500 platform (Illumina Inc., CA, USA).

### Hybrid Sequencing Data Analysis

The raw SMRT sequencing data were processed with SMRT Link 6.0 software (https://www.pacb.com/support/software-downloads) to select suitable subreads with lengths ≥ 50 bp and read scores ≥ 0.65. Then, the suitable subreads were used to select circular consensus sequences (CCSs) with pass values ≥ 1 and predicted accuracy values ≥ 0.8. CCSs with a 5´ primer, 3´ primer, and poly A tail were classified as a full-length read. Consensus reads were then obtained using iterative clustering for error correction (ICE), and Quiver was applied to further polish the consensus reads that could produce high-quality consensus reads (length > 200, high-quality > 0.99) (Eid et al., 2009). Finally, high-quality and nonredundant isoforms were obtained by using CD-HIT to filter redundant isoforms (Li and Godzik, 2006).

The raw reads from Illumina sequencing were processed using in-house Perl scripts to obtain clean data (PRJNA559687). High-quality clean reads did not contain adaptors or poly(-N) sequences. Then, the nucleotide errors of the SMRT high-quality consensus reads were corrected using the clean Illumina data with LoRDEC (http://atgc.lirmm.fr/lordec) software (parameter: -k 23; -s 3) (Salmela and Rivals, 2014).

### Reads Map to Reference Genome and Gene Function Annotation

In this study, we used GMAP (http://research-pub.gene.com/gmap/) software to map the corrected consensus reads to the reference genome with the following parameters: no-chimeras; -n 1 (Wu and Watanabe, 2005). A portion of the corrected consensus reads were mapped in the un-annotated region in the reference genome, and we defined these reads as novel genes. Moreover, to identify the functions of all genes, the Gene Ontology (GO) database, Kyoto Encyclopedia of Genes and Genomes (KEGG) database, EuKaryotic Orthologous Groups (KOG) database, nucleotide (NT) database, nonredundant (NR) database, Protein Family (Pfam) database, and SWISS-PROT database, were used to annotate all genes (Kanehisa and Goto, 1999; Tatusov et al., 2003; Pruitt et al., 2007; Young et al., 2010; Finn et al., 2016; The UniProt, 2017). The NR, KOG, SWISS-PROT, and KEGG database annotation was performed on Diamond blastx (https://github.com/bbuchfink/diamond) software (parameters: more-sensitive –k 10; -e 1e-5; -f 6; -p 4) (Buchfink et al., 2015); NT database annotation was performed on blastn (ftp://ftp.ncbi.nlm.nih.gov/blast/executables/blast+/LATEST/) software (parameters: -outfmt 6; -evalue 1e-5; -max_target_seqs 10; -num_threads 4) (Altschul et al., 1997); and Pfam database annotation was performed on Hmmscan (http://hmmer.org/download.html) software (parameters: –acc; –domtblout) (Finn et al., 2011).

### Identification of Differentially Expressed Genes

Cuffdiff (http://cole-trapnell-lab.github.io/cufflinks/) software was used to calculate the fragments per kilobase of transcript sequence per million mapped reads (FPKM) values of all transcripts (parameters: FDR 0.05; library-norm-method geometric; dispersion-method pooled) (Sreya and Chon-Kit Kenneth, 2016). Based on the FPKM values, we used DESeq2 (http://www.bioconductor.org/packages/release/bioc/html/DESeq.html) software to identify the differentially expressed genes with adjusted P-values smaller than 0.05.

### Identification of AS Events and GO/KEGG Enrichment Analyses of AS Genes

In this study, AS events were identified using SUPPA (https://bitbucket.org/regulatorygenomic-supf/suppa) software with the default parameters (Abdel-Ghany et al., 2016). The SUPPA software classified basic AS events into seven types: IR, mutual exon exclusion (MX), alternative 5′ splicing (A5), alternative 3′ splicing (A3), alternative first exon splicing (AF), alternative last exon splicing (AL), and ES (**Supplementary Material 1**). To better understand the functions of AS genes and relationships with their products, we performed GO/KEGG enrichment analyses using GOseq software (http://www.bioconductor.org/packages/release/bioc/html/goseq.html) and KOBAS software (http://kobas.cbi.pku.edu.cn/download.php). GO terms and KEGG terms with corrected P-values less than 0.05 were considered significantly enriched terms.

### Identification of Alternatively Spliced lncRNAs

In this study, we used the Coding Potential Calculator (CPC, http://cpc.cbi.pku.edu.cn/), the Coding-Non-Coding Index (CNCI, https://github.com/www-bioinfo-org/CNCI), the Pfam database, and the Predictor of Long noncoding RNAs and mEssenger RNAs based on an improved K-mer scheme (PLEK, https://sourceforge.net/projects/plek/) to identify lncRNAs with the default parameters (Kong et al., 2007; Sun et al., 2013; Li. et al., 2014; Finn et al., 2016). Transcripts were identified as putative lncRNAs only if they were predicted to be lncRNAs by all four tools. Then we used SUPPA software to identify lncRNAs that underwent AS events.

### WGCNA Analysis and Hub Gene Identification

A WGCNA package in R software was used to construct the AS gene coexpression network. Only genes ranked in the top 60% based on the ranking of the variance of FPKM values could be used for the WGCNA analysis. The pickSoftThreshold function was applied to calculate the soft threshold power, and we selected the most suitable power (16 in this study) for gene coexpression network construction. The gene coexpression network was constructed using the blockwiseModules function with the following parameters: the power was 16, the TOM type was signed, and the min module size was 300, and the other parameters were the default values.

For hub gene identification, the degree algorithm in the CytoHubba package of Cytoscape software (http://www.cytoscape.org/download.html) was used to identify hub genes in the six modules based on the WGCNA analysis results. In each module, only the top 10% genes in the degree algorithm were considered as hub genes. Hub genes and their related gene were selected for network visualization using Gephi software (https://gephi.org/).Validation of AS events using reverse transcription PCR

To validate whether AS events truly occurred, we randomly selected 8 AS genes and designed specific PCR primers using Oligo 7 software (https://en.freedownloadmanager.org/Wind-owsPC/OLIGO.html). All primers are shown in **Supplementary Material 2**. After synthesizing cDNA using the PrimeScript™ RT Master Mix (TaKaRa, Dalian, China), 20 μl of cDNA was diluted 10 times. Then, 2 μl of diluted cDNA was used as the template for PCR according to the instructions of a Phanta^®^ Max Super-Fidelity DNA Polymerase Kit (Vazyme, Nanjing, China). The PCR procedure was as follows: 95°C pre-denaturation for 3 min; 30 cycles of denaturation (95°C for 15 s), annealing (57.5–62.5°C for 15 s), and elongation (72°C, elongation rate of 1 kb per min); and 72°C complete elongation for 5 min. Finally, 1.5 percent (w/v) agarose gel electrophoresis was used to verify the amplification result.

## Results

### Overview of the Transcriptome and AS in *L. chinense*

**Figure 1:**
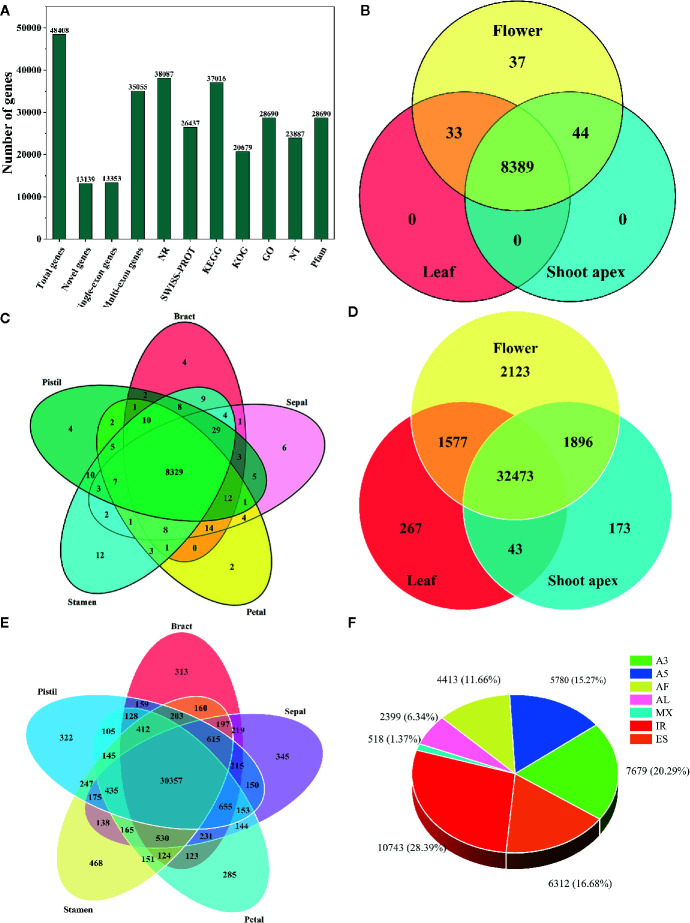
Information about the transcriptome and alternative splicing (AS) in *L. chinense*. **(A)** Statistics for genes and their annotation in seven databases. **(B)** Distribution of alternatively spliced genes in flower, leaf and shoot apex tissues. **(C)** Distribution of alternatively spliced genes in pistil, stamen, petal, sepal and bract tissues. **(D)** Distribution of alternatively spliced transcripts in flower, leaf and shoot apex tissues. **(E)** Distribution of alternatively spliced transcripts in pistil, stamen, petal, sepal and bract tissues. **(F)** The proportions of seven AS events in *L. chinense*.

Using hybrid sequencing, we detected a total of 48,408 genes, including 13,139 novel genes (**Figure 1A**). Among these genes, 13,353 had a single exon while 35,055 had multiple exons (**Figure 1A**). Moreover, upon conducting functional annotation with seven databases, we found that the NR database annotated the highest number of genes (38,087), followed by the KEGG (37,016), GO (28,690), Pfam (28,690), SWISS-PROT (26,437), NT (23,887) and KOG (20,679) databases (**Figure 1A**).

Through analysis with the SUPPA tool, we found that only 8,503 genes (210 single-exon genes and 8,293 multi-exon genes) underwent AS, and most AS genes (8,389) could be found simultaneously in leaf, flower and shoot apex tissues while 37 AS genes were found only in flowers (**Figure 1B**). In pistils, stamens, petal, sepals and bracts, we found 4, 12, 2, 6, and 4 specifically expressed AS genes, respectively (**Figure 1C**). Intriguingly, we found that the number of tissue-specific AS transcripts was greater than the number of tissue-specific AS genes. Among the 2,123 tissue-specific AS transcripts, only 267 and 173 tissue-specific AS transcripts were detected in leaves and shoot apices, respectively (**Figure 1D**). Focusing on flower tissues, stamens had the largest number of tissue-specific AS variants (468), followed by sepals (345), pistils (322), bracts (313) and petals (285) (**Figure 1E**). Moreover, we identified 37,844 AS events from these 8,503 AS genes (**Figure 1F**). The most common AS event was IR (10,743, 28.39%), followed by A3 (7,679, 20.29%), ES (6,312, 16.68%), A5 (5,780, 15.27%), AF (4,413, 11.66%), and AL (2,399, 6.34%), while MX (518, 1.37%) was the least common AS event (**Figure 1F**). In other plants, such as peanut, tomato, and moso bamboo, IR has been found to be the predominant AS event (Sun and Xiao, 2015; Ruan et al., 2018; Zhao et al., 2018). Thus, IR may have a great influence in the transcriptome complexity of *L. chinense*.

### GO and KEGG Enrichment Analyses of AS Genes

**Figure 2:**
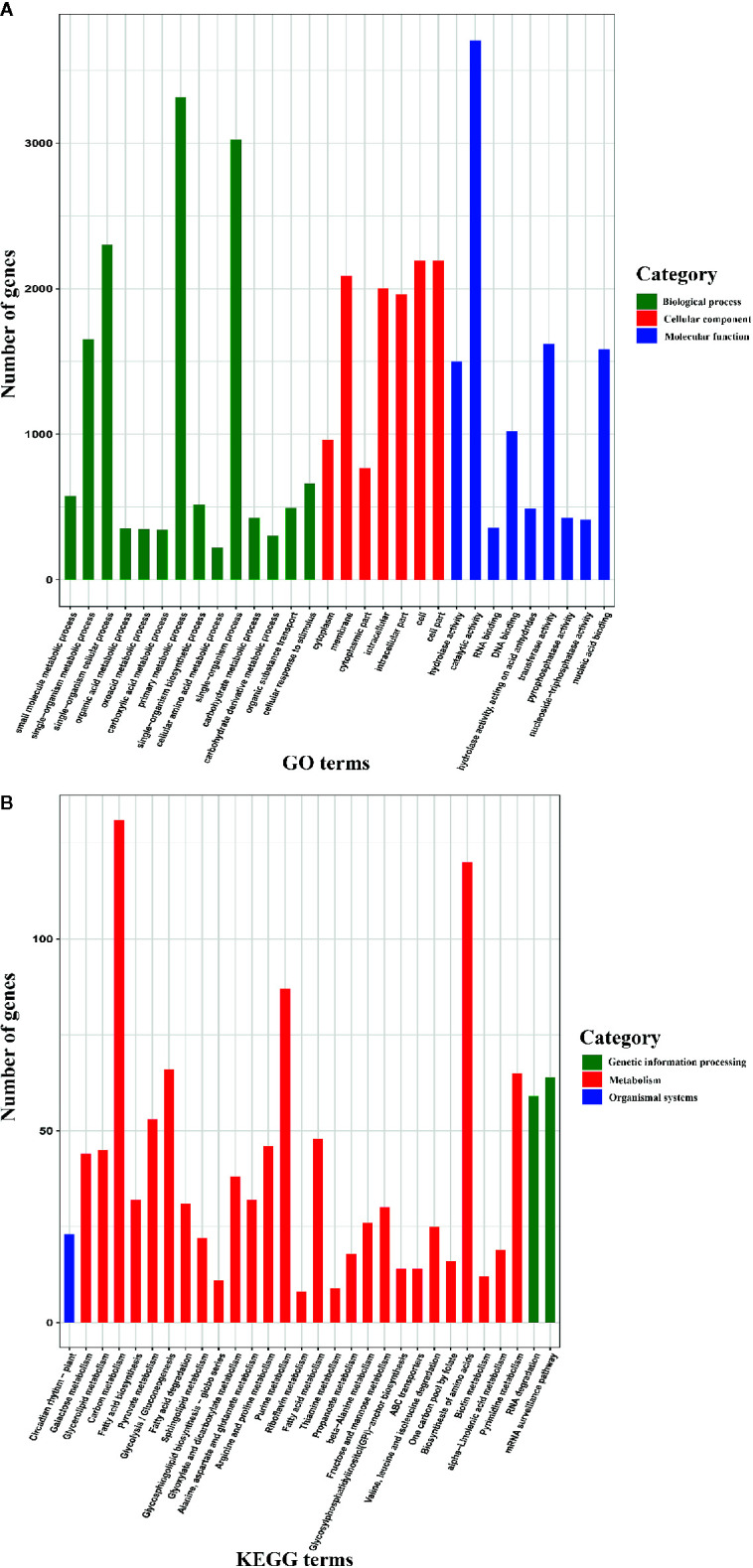
Gene Ontology (GO) database and Kyoto Encyclopedia of Genes and Genomes (KEGG) enrichment analyses of alternative splicing (AS) genes (only the top 30 terms are shown). **(A)** GO enrichment analysis of AS genes. **(B)** KEGG pathway enrichment analysis of AS genes.

To further understand the functions of AS genes, we conducted GO and KEGG enrichment analyses of 8,503 AS genes. The GO enrichment analysis revealed that the top six terms were “catalytic activity”, “primary metabolic process”, “single-organism process”, “single-organism cellular process”, “cell”, and “cell part” (**Figure 2A**), indicating that these AS genes play roles in basic physiological and biochemical processes. Moreover, the KEGG pathway enrichment analysis revealed that these AS genes participated in 121 pathways, most of which are associated with metabolism, such as carbon metabolism, purine metabolism, and pyrimidine metabolism (**Figure 2B**). In plants, AS plays important roles in regulating growth and development, flowering, the stress response, and other biological processes (Filichkin et al., 2015; Szakonyi and Duque, 2018). Therefore, unsurprisingly, the AS genes participated in multiple pathways and were associated with multiple biological functions.

### Expression Levels of AS Genes and Splice Variants

**Figure 3:**
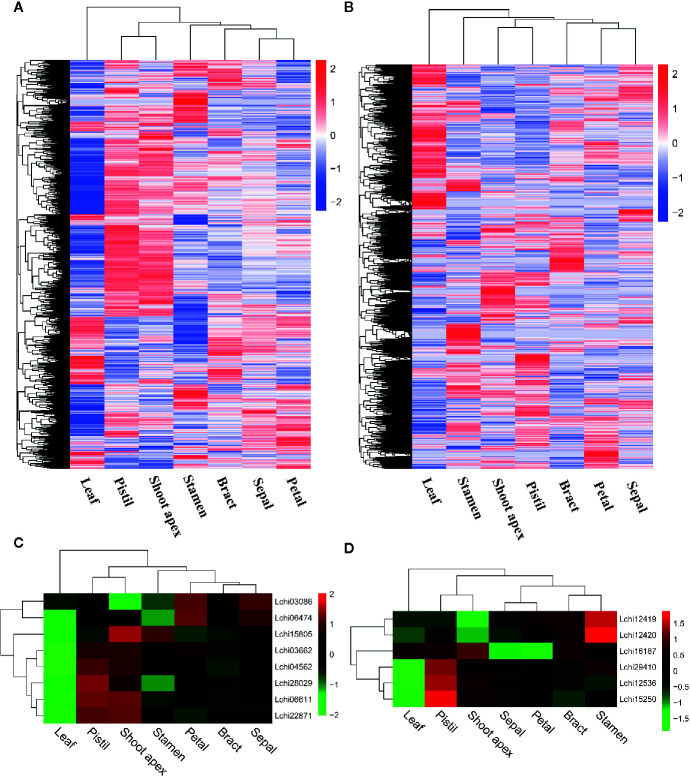
Hierarchical clustering analysis and the relative expression profile of genes in different tissues of *L. chinense*. Hierarchical clustering analysis of alternative splicing (AS) genes **(A)** and alternatively spliced variants **(B)** in seven tissues. Differentially expressed serine/arginine-rich (SR) protein genes **(C)** and heterogeneous nuclear ribonucleoprotein (hnRNP) genes **(D)** in seven tissues.

To better understand the expression levels of AS genes and splice variants in different tissues, we performed a hierarchical clustering analysis. We found that most AS genes were strongly expressed in pistil and shoot apex tissues but weakly expressed in leaf tissues (**Figure 3A**). However, many AS genes were strongly expressed in leaves but weakly expressed in pistils, shoot apices, and stamens (**Figure 3A**). When we analyzed the AS variant levels, we found a different pattern from that observed for the AS gene levels. A large proportion of AS variants were highly expressed in leaves but weakly expressed in pistils, shoot apices, and stamens (**Figure 3B**). A gene can produce multiple splice variants through AS, and the expression levels of splice variants produced by the same gene can be different in different tissues (Sun and Xiao, 2015). Thus, the differences in the expression levels of AS genes and splice variants were unexpected.

### Differentially Expressed SR Genes and hnRNP Genes

SR proteins and hnRNPs play important roles in regulating constitutive splicing and AS (Vitulo et al., 2014). To identify the SR genes and hnRNP genes that may affect AS, we analyzed the expression levels of such genes in seven tissues of *L. chinense*. Ultimately, we detected eight differently expressed SR genes and six hnRNP genes (**Figures 3C, D**). Intriguingly, all eight SR genes were expressed at low levels in leaves while two SR genes (*Lchi03086* and *Lchi06474*) were highly expressed in petals and sepals, five SR genes (*Lchi03662*, *Lchi04562*, *Lchi28029*, *Lchi06611*, and *Lchi22871*) were highly expressed in pistils, and six SR genes (*Lchi15805*, *Lchi03662*, *Lchi04562*, *Lchi28029*, *Lchi06611*, and *Lchi22871*) were highly expressed in shoot apices (**Figure 3C**). Among the six differentially expressed hnRNP genes, one (*Lchi16187*) was strongly expressed in leaves; the other five hnRNP genes (*Lchi12419*, *Lchi12420*, *Lchi29410*, *Lchi12536*, and *Lchi15250*) were weakly expressed in leaves, and four of them (*Lchi16187*, *Lchi29410*, *Lchi12536*, and *Lchi15250*) were also highly expressed in pistils and shoot apices (**Figure 3D**). Notably, most of the differentially expressed SR and hnRNP genes were expressed at low levels in leaves and at high levels in pistils and shoot apices; these findings were consistent with the expression patterns of most AS genes in leaves, pistils and petals (**Figures 3A, C, D**). We speculated that these differentially expressed SR and hnRNP genes may play important roles in AS regulation of *L. chinense*.

### IR Affects AS Transcripts

**Figure 4:**
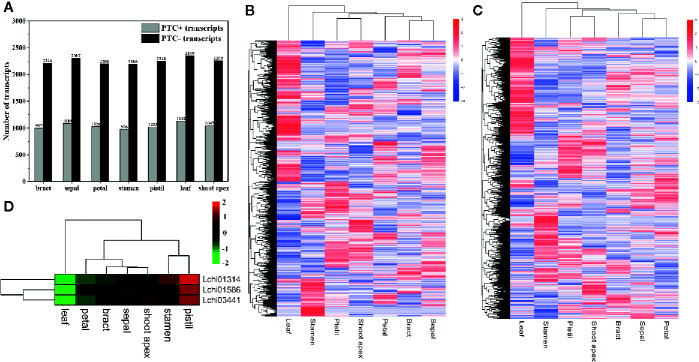
Statistics and hierarchical cluster analysis of PTC^+^ transcripts, PTC^-^ transcripts, and Upf genes. The log_10_(FPKM + 1) values of transcripts were used for the hierarchical cluster analysis **(A)** Statistics of PTC^+^ transcripts and PTC^-^ transcripts in different tissues. **(B, C)** Hierarchical cluster analysis of PTC^+^ transcripts and PTC^-^ transcripts, respectively. **(D)** Hierarchical cluster analysis of five Upf genes in *L. chinense*.

As the most common AS event in plants, IR can make genes produce nonproductive transcripts by introducing PTCs into the coding sequence, and transcripts with PTCs can be degraded by the NMD pathway; thus, this process affects the proteome diversity (Marquez et al., 2012; Vitulo et al., 2014; Filichkin et al., 2018). To investigate how many PTC-containing (PTC^+^) transcripts and non-PTC-containing (PTC^-^) transcripts were produced due to IR, we analyzed the number of PTC^+^ transcripts and PTC^-^ transcripts in seven tissues. Firstly, we filtered the transcripts with IR that had FKPM values less than 1 in all tissues. Then, we identified a total of 1,656 PTC^+^ transcripts, among which the majority were detected in leaves (1,132) and few were present in stamens (976) (**Figure 4A**). A total of 555 PTC^+^ transcripts were present in all seven tissues, and 117, 64, 49, 36, 25, 23, and 15 tissue-specific PTC^+^ transcripts were detected in leaves, stamens, shoot apex, pistils, petals, sepals, and bracts, respectively (**Supplementary Material 3, Figure S2A**). Moreover, we identified a total of 3,310 PTC^-^ transcripts; the number of PTC^-^ transcripts in seven tissues ranged from 2,190 (stamens) to 2,349 (leaves) (**Figure 4A**). Moreover, 1,383 PTC^-^ transcripts existed in all seven tissues, and the 513 tissue-specific PTC^-^ transcripts included 188 leaf-specific, 108 stamen-specific, 70 pistil-specific, 56 shoot-apex-specific, 33 bract-specific, 32 petal-specific, and 26 sepal-specific PTC^-^ transcripts (**Supplementary Material 3, Figure S2B**). Overall, we found that the ratio between the PTC^+^ transcript number and PTC^-^ transcript number was approximately one to two (**Figure 4A**). Through the hierarchical cluster analysis, we found that a large number of transcripts, including both PTC^+^ transcripts and PTC^-^ transcripts, were highly expressed in leaves (**Figures 4B, C**).

Recent studies show that NMD regulates approximately 10% of mRNAs by downregulating them to meet the demands of organisms, and during this process three up-frameshift (Upf) proteins (Upf1, Upf2, and Upf3) play pivotal roles (Karousis et al., 2016; Gupta and Li, 2018). To determine the potential relationship between Upf proteins and PTC^+^ transcripts at the expression level, we identified three Upf homologous genes (*Lchi01314* [Upf1], *Lchi03441* [Upf2], and *Lchi01586* [Upf3]) in *L. chinense* and confirmed their expression profile. We found that these three Upf homologous genes tended to highly expressed in pistils, sepals, stamens, and shoot apices but lowly expressed in leaves, bracts, and petals (**Figure 4D**). Especially in pistils and leaves, the expression profile of the three Upf homologous genes in these two tissues was obviously opposite (**Figure 4D**). However, we could not find a stronger relationship between Upf genes and PTC^+^ transcripts at the expression level. Notably, not all PTC^+^ transcripts are targeted by NMD (Feng et al., 2015). We speculated that the low proportion of PTC^+^ transcripts targeted by NMD may mask the relationship between Upf genes and PTC^+^ transcripts at the expression level.

### WGCNA Analysis of AS Genes and Hub Gene Identification

**Figure 5:**
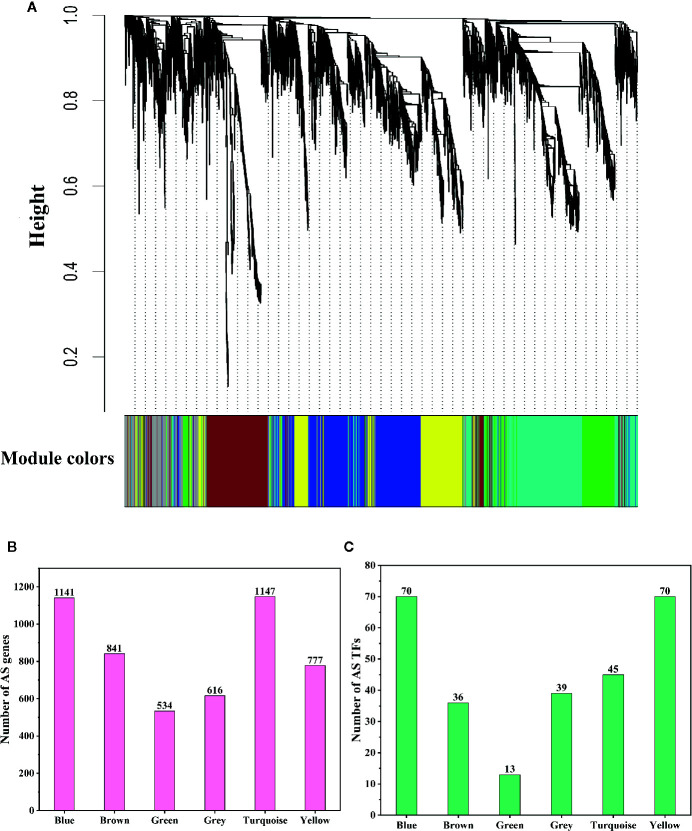
Dendrogram of coexpressed alternative splicing (AS) genes and statistics of AS gene numbers and AS transcription factor (TF) numbers. **(A)** Dendrogram of coexpressed AS genes in *L. chinense*. The genes and coexpression modules are represented by vertical lines (upper panel) and colored rectangles (bottom panel), respectively. **(B)** Numbers of AS genes in six coexpression modules. **(C)** Numbers of AS TFs in six coexpression modules.

**Figure 6:**
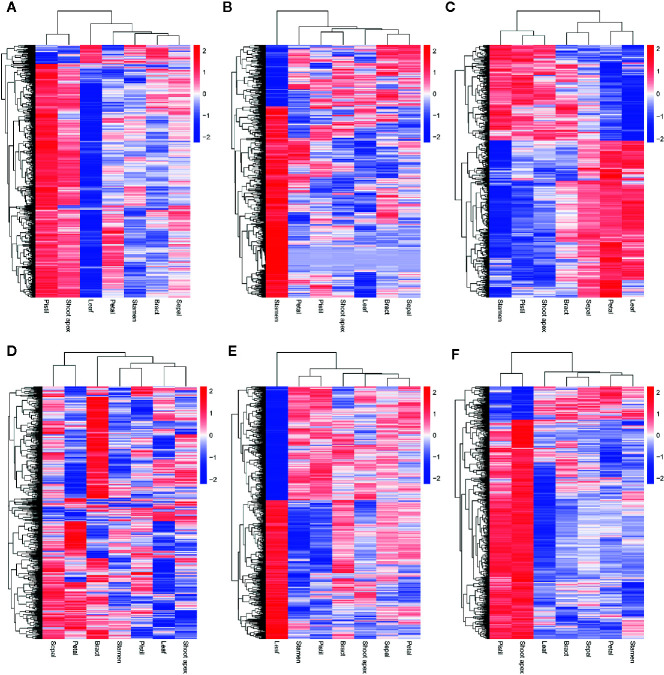
Hierarchical cluster analysis of coexpressed alternative splicing (AS) genes in six modules. The log_10_(FPKM + 1) values of AS genes were used for the analysis. **(A–F)** Hierarchical cluster analysis of coexpressed AS genes in the blue, brown, green, gray, turquoise, and yellow modules, respectively.

Although we found that most AS genes, SR genes, hnRNP genes, and Upf genes had opposite expression patterns between leaves and pistils, we could not identify the relationship between these patterns. To solve this problem, we performed WGCNA analysis of the AS genes, SR genes, hnRNP genes, and Upf genes. To improve the reliability of the analysis results, we selected the AS genes for which the rank of the variance of the FPKM value was in the top 60% for analysis. In total, 5,056 genes were classified into 6 coexpression modules, among which 1,141; 841; 534; 616; 1,147, and 777 AS genes were classified into the blue, brown, green, gray, turquoise, and yellow modules, respectively (**Figures 5A, B**). We also found that 70, 36, 13, 39, 45, and 70 AS transcription factors (TFs) were classified into the blue, brown, green, gray, turquoise, and yellow modules, respectively (**Figure 5C**). Then, we performed a hierarchical cluster analysis of these AS genes in the six coexpression modules. We found that AS genes in different modules showed obviously different expression patterns (**Figure 6**). Most AS genes from the blue module were strongly expressed in the pistils and shoot apices but weakly expressed in the leaves (**Figure 6A**). AS genes in the yellow module also showed similar expression patterns (**Figure 6F**). Moreover, AS genes in the brown module were highly expressed in the stamens, and AS genes from the gray module tended to be highly expressed in the bracts (**Figures 6B, D**). However, a large number of AS genes from the turquoise module showed high expression levels in the leaves but presented low expression levels in the stamens and pistils (**Figure 6E**). AS genes in the green module were clustered into two subclusters. One subcluster showed high expression patterns in the stamens, pistils, shoot apices, and bracts, while the other subcluster showed high expression patterns in the sepals, petals, and leaves (**Figure 6C**).

To determine what functions these AS genes play, we carried out GO and KEGG enrichment analysis. In the results of the GO enrichment analysis, we found that although the expression patterns of blue-module AS genes and yellow-module AS genes were very similar, there were obvious differences between them in the GO enrichment analysis results. For example, blue-module AS genes were significantly enriched in 12 cellular component terms, but yellow-module AS genes were not significantly enriched in cell component terms (**Supplementary Material 4, Figures S3A, F** **)**. Moreover, blue-module AS genes were significantly enriched in transcription, post-transcription regulation, and translation related terms, such as translation, gene expression, ribosome biogenesis, RNA binding, DNA binding, and nucleic acid binding (**Supplementary Material 4, Figure S3A**). AS genes in the brown module were significantly enriched in carbohydrate metabolism-related terms, such as carbohydrate metabolic processes, hydrolase activity, and galactosidase activity (**Supplementary Material 4, Figure S3B**). AS genes from the green module were significantly enriched in multiple metabolic process (**Supplementary Material 4, Figure S3C**). However, AS genes in the gray module were only significantly enriched in six terms (**Supplementary Material 4, Figure S3D**). Moreover, AS genes in the turquoise module were significantly enriched in cytoplasm-related and oxidoreductase activity-related terms (**Supplementary Material 4, Figure S3E**). According to the KEGG pathway enrichment analysis results, only AS genes in the blue, brown, green, and turquoise modules were significantly enriched (**Supplementary Material 5, Figure S4**). It seemed that blue-module AS genes participated in transcription, translation, and post-transcriptional regulation, because they were significantly enriched in RNA transport, RNA polymerase, spliceosome, ribosome, and mRMA surveillance pathways (**Supplementary Material 5, Figure S4A**). We also found that AS genes from the brown module and green module were significantly enriched in multiple metabolism pathways, such as sphingolipid metabolism, glycerolipid metabolism, pyruvate metabolism, and fatty acid metabolism (**Supplementary Material 5, Figures S4B, C**). Moreover, we determined that AS genes from the turquoise module participate in photosynthesis, because they were significantly enriched in the glyoxylate and dicarboxylate metabolism pathway and carbon metabolism pathway (**Supplementary Material 5, Figure S4E**).

**Figure 7:**
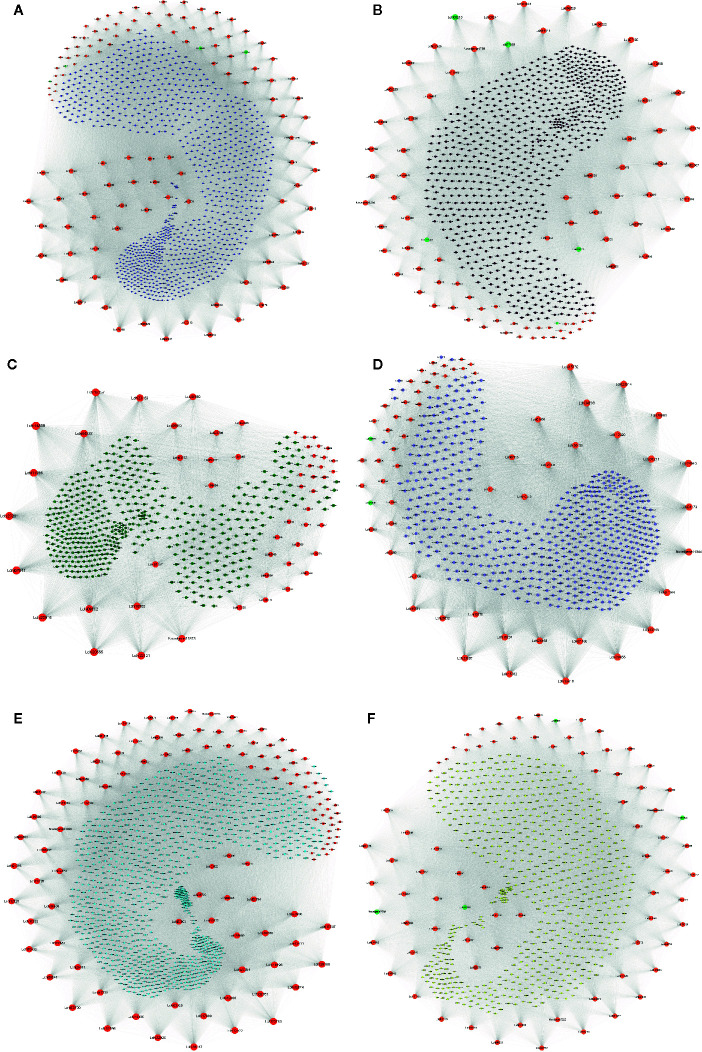
Network of hub alternative splicing (AS) genes and their related genes in six coexpression modules. Hub genes are represented by orange-red notes, medium-aquamarine notes represent TFs that were also identified as hub genes, and other colored notes represent genes that were related to hub genes. The size of the note represents the degree of interaction between the hub gene and other genes, and larger notes indicate that the hub gene interacts with more genes. **(A–F)** Network of hub AS genes and their related genes in the blue, brown, green, gray, turquoise, and yellow modules, respectively.

To identify the hub genes that may play pivotal roles in different pathways, the degree algorithm in the CytoHubba package of Cytoscape software was applied. The networks of hub genes and their related genes in six coexpression modules were constructed and are shown in **Figure 7**. In the blue module, we identified 114 hub genes, among which 21 hub genes were involved in 7 significantly enriched KEGG pathways. *Lchi21288* (GTP binding protein) was related to the largest number of genes (1,121) (**Figure 7A**). Four AS TFs were identified as hub genes in the blue module, which were *Lchi01126* (FAR1 family), *Lchi29242* (C2H2 family), *Lchi05152* (C2H2-GATA family), and *Lchi21823* (C2H2 family) (**Figure 7A**). In the brown module, 84 hub genes were identified, including 5 AS TFs—*Lchi06210* (bZIP family), *Lchi27955* (MYB family), *Lchi26255* (MADS-M-type family), *Lchi11540* (bHLH family), and *Lchi17316* (NAC family)—and *Lchi12869* (Delta-1-pyrroline-5-carboxylate synthase), which were related to the largest number of genes (837). Notably, *Lchi10151* (Alpha-galactosidase 1) was involved in 4 significantly enriched KEGG pathways (**Figure 7B**). For the green module, 53 hub genes were identified, and *Lchi14809* (peptide chain release factor PrfB1) was associated with the largest number of genes (516); however, only 2 hub genes were participated in 1 significantly enriched KEGG pathway (**Figure 7C**). In the network of the gray module, 62 AS genes were considered as hub genes, including 2 AS TFs—*Lchi22536* (CAMTA family) and *Lchi00208* (CPP family)—and *Lchi30455* (LRR receptor-like serine/threonine-protein kinase), which had a relationship with the maximum number of genes (613) (**Figure 7D**). For the turquoise module, we identified 115 hub genes, among which 18 hub genes participated in 3 significantly enriched KEGG pathways, among which *Lchi20763* (kynurenine–oxoglutarate transaminase 1-like protein), *Lchi29168* (epimerase family protein SDR39U1 homolog), and *Lchi13522* (photosynthetic NDH subunit of subcomplex B 5) were the top three hub genes related to 1,115, 1,084, and 1,073 genes, respectively (**Figure 7E**). In the yellow module network, 78 hub genes were identified, including 4 AS TFs—*Lchi00996* (MYB family), *Lchi07039* (C3H family), *Lchi08842* (MYB family), and *Novelgene2009* (bHLH family)—and *Lchi34059* (protein IQ-DOMAIN), which was associated with the largest number of genes (752) (**Figure 7F**).

We could deduce from the above results that blue-module genes related to gene expression and post-transcriptional regulation are strongly expressed in pistils but weakly expressed in leaves, leading to differences in the activities of gene expression and post-transcriptional regulation between these two tissues and further resulting in genes related to AS, such as SR genes, hnRNP genes, and even the Upf gene, showing opposite expression patterns in pistils and leaves. Moreover, we also found that AS affects multiple physiological and biochemical processes in *L. chinense*, such as photosynthesis, sphingolipid metabolism, fatty acid biosynthesis and metabolism. Furthermore, AS TFs were identified as hub genes in four coexpression modules, and it seemed that AS could influence *L. chinense* by influencing TFs.

### Differences Between AS Genes and Non-AS Genes

**Figure 8:**
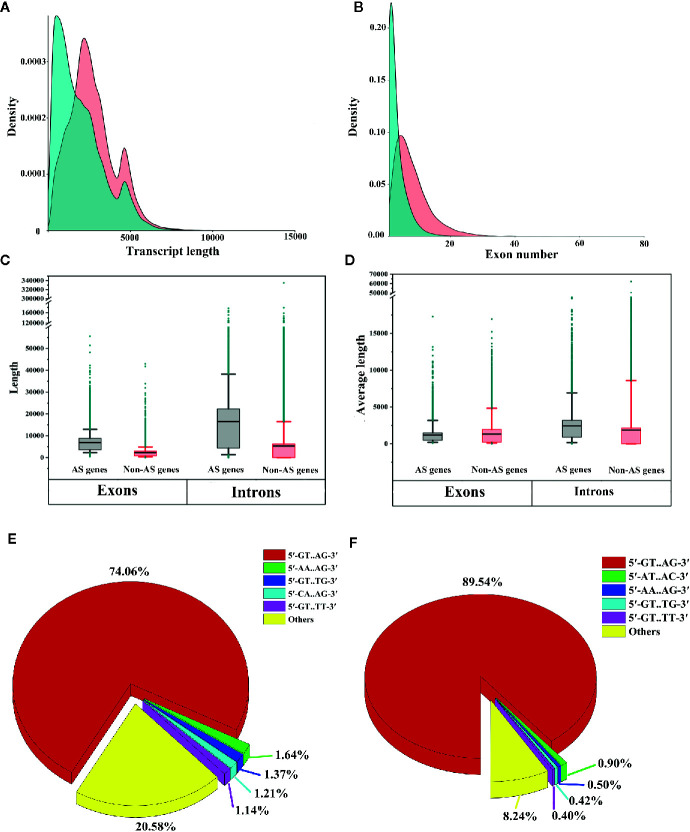
Comparative analysis of alternative splicing (AS) genes and non-AS genes. **(A)** Comparison of transcript lengths between AS genes and non-AS genes. **(B)** Comparison of the numbers of exons of AS genes and non-AS genes. **(C)** Box plot of exon lengths and intron lengths of AS genes and non-AS genes. **(D)** Box plot of the average lengths of exons and introns of AS genes and non-AS genes. Boxes represent 25^th^ and 75^th^ percentiles. Whiskers represent 5^th^ and 95^th^ percentiles, and black lines in the boxes represent the median values. Dark cyan circles represent outliers. **(E)** Proportion of splicing sites in AS genes (only shown top 5 types). **(F)** Proportion of splicing sites in non-AS genes (only the top 5 types are shown).

To identify the differences between AS genes and non-AS genes, we compared the transcript lengths, exon numbers, total exon lengths, total intron lengths, average exon lengths and average intron lengths between AS genes and non-AS genes. We found that AS genes tended to produce longer transcripts and have more exons compared to non-AS genes (**Figures 8A, B**). We also noticed that the total lengths of exons and introns of AS genes were longer than those of non-AS genes (**Figure 8C**). On average, the exons of AS genes were shorter than those of non-AS genes, while the introns of AS genes were longer than those of non-AS genes (**Figure 8D**). This indicates that the structural feature of genes may affect AS.

To investigate whether differences existed in splicing sites between AS genes and non-AS genes, we extracted two nucleotides from the ends of introns and analyzed the proportions of different splicing sites in the genes. We observed that for both AS genes and non-AS genes, canonical splicing sites (5′-GT.AG-3′) were predominant (70.46% for AS genes and 89.54% for non-AS genes) (**Figures 8E, F**). However, noncanonical splicing sites, such as 5′-AA.AG-3′, 5′-GT.TG-3′, and 5′-GT.TT-3′, accounted for only small proportions of the splicing sites in AS genes and non-AS genes (**Figures 8E, F**). These results are consistent with findings obtained for tomato and *Ananas comosus* var. *bracteatus* (Clark et al., 2019; Ma et al., 2019).

### Relationships Among AS Frequency, AS Events, and Gene Features

**Figure 9:**
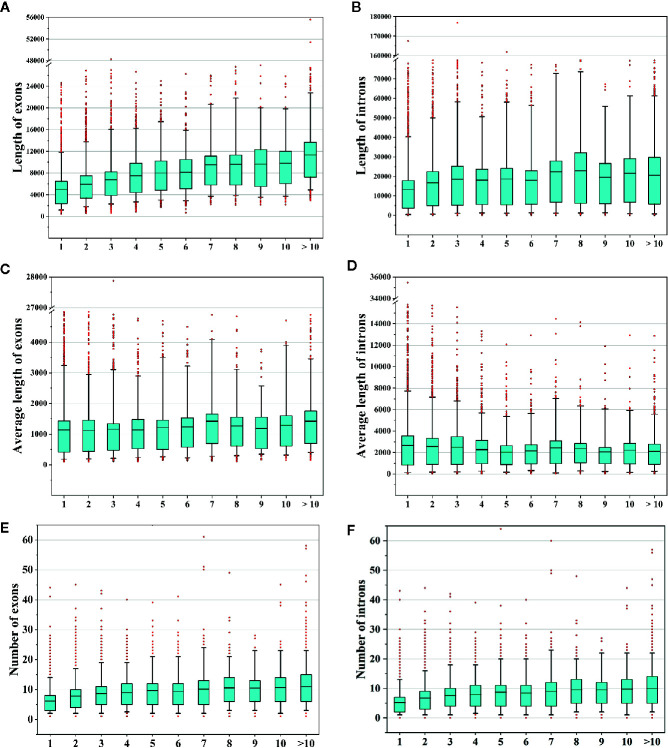
Analysis of alternative splicing (AS) events and gene features. **(A, B)** Comparison of the total lengths of exons/introns of AS genes that underwent different numbers of AS events. **(C, D)** Comparison of the average lengths of exons/introns of AS genes that underwent different numbers of AS events. **(E, F)** Comparison of the numbers of exons/introns of AS genes that underwent different numbers of AS events.

In this study, we identified 37,844 AS events from 8,503 AS genes (**Figure 1F**), indicating that multiple AS events occurred for a single gene. We next sought to determine whether the frequencies of AS events were related to gene features such as the numbers and lengths of exons/introns. Therefore, we compared the total lengths of exons/introns, the average lengths of exons/introns, and the numbers of exons/introns among AS genes with different AS event frequencies. We found that the total exon lengths and total intron lengths of AS genes increased with increasing AS frequencies (**Figures 9A, B**). However, when we compared the average lengths of exons/introns, we found that the relationship between the AS event frequency and the average exon/intron length was not strong (**Figures 9C, D**). In addition, the number of exons or introns increased with increasing AS event frequencies (**Figures 9E, F**). These findings suggest that genes with more complex structures tend to undergo more AS events.

**Figure 10:**
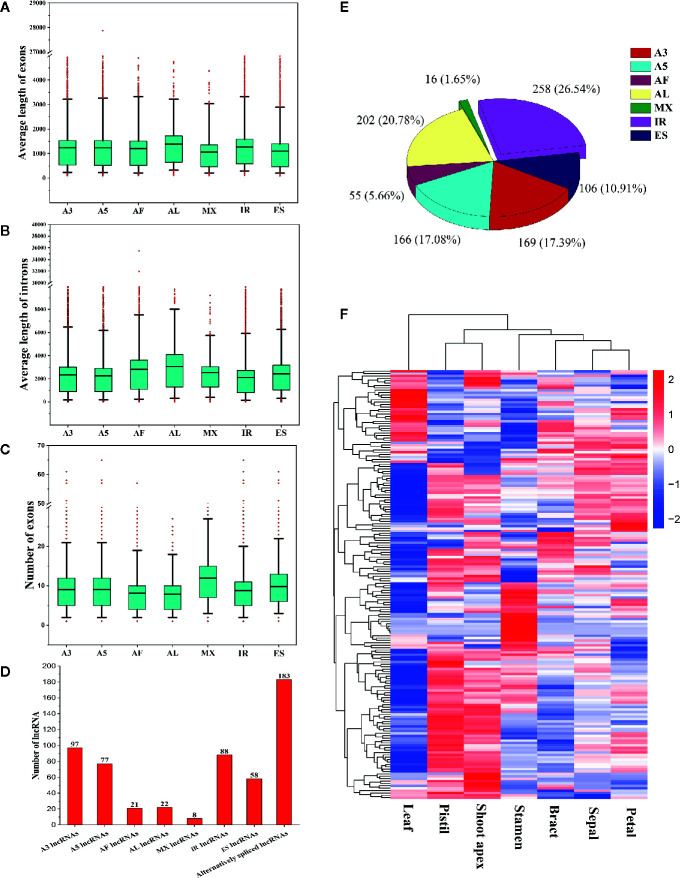
Comparison of different alternatively spliced genes and information about alternatively spliced lncRNAs. **(A)** Comparison of the average exon lengths of seven types of alternative splicing (AS) genes. Boxes represent 25^th^ and 75^th^ percentiles. Whiskers represent 5^th^ and 95^th^ percentiles, and black lines in the boxes represent the median values. Red circles represent outliers. The X-axis represents genes undergoing different types of AS events. **(B)** Comparison of the average intron lengths of seven types of AS genes. **(C)** Comparison of exon numbers in seven types of AS genes. **(D)** Statistics for the numbers of different types of alternatively spliced long noncoding RNAs (lncRNAs). **(E)** The proportions of seven types of AS events occurring in lncRNAs. **(F)** Hierarchical clustering analysis of alternatively spliced lncRNAs.

To determine whether gene features were related to AS event types, we compared the average lengths of exons/introns and the numbers of exons among AS genes undergoing different types of AS events. We found that genes that underwent AL events had the longest average lengths of exons and introns (**Figures 10A, B**). Genes that underwent MX events or ES events had the shortest average exon lengths, and genes that underwent IR had the shortest average intron lengths (**Figures 10A, B**). Interestingly, genes that underwent AL events had the fewest exons, whereas genes that underwent MX events had the most exons (**Figure 10C**). These results indicate that shorter exons tend to be skipped, shorter introns tend to be retained, and genes with more exons tend to experience MX events. These phenomena are also observed in moso bamboo (Wang et al., 2018).

### Identification of Alternatively Spliced lncRNAs

In this study, we identified 183 alternatively spliced lncRNAs (**Figure 10D**). Among these lncRNAs, 97 underwent A3 events, 88 underwent IR events, 77 underwent A5 events, 58 underwent ES events, 22 underwent AL events, 21 underwent AF events, and 8 underwent MX events (**Figure 10D**). A total of 972 AS events occurred in the 183 alternatively spliced lncRNAs (**Figure 10E**). IR events (258, 26.54%) constituted the largest proportion of AS events in lncRNAs, followed by AL (202, 20.78%), A3 (169, 17.39%), A5 (166, 17.08%), and ES (106, 10.91%) events, whereas MX events (16, 1.65%) constituted the smallest proportion of AS events in lncRNAs (**Figure 10E**). Through the hierarchical clustering analysis, we found that most alternatively spliced lncRNAs were preferentially highly expressed in shoot apices, stamens, and pistils (**Figure 10F**). Interestingly, the expression patterns of alternatively spliced lncRNAs appeared to be similar to those of the differently expressed SR proteins and hnRNP genes (**Figure 3**), indicating that SR proteins and hnRNP genes may substantially influence the production of alternatively spliced lncRNAs.

### Validation of AS Events Using PCR

**Figure 11:**
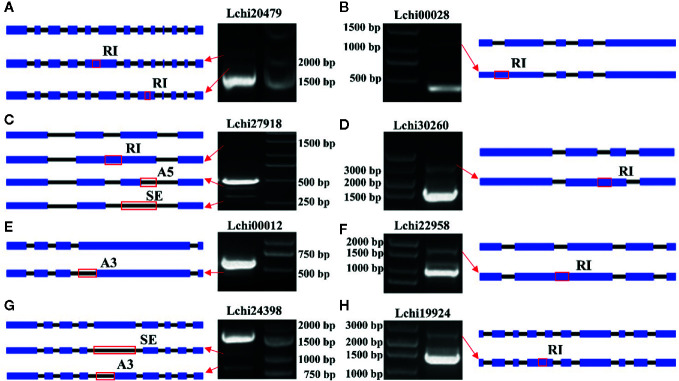
Validation of alternative splicing (AS) events in eight AS genes. **(A–H)** Gene structure and polymerase chain reaction (PCR) results of *Lchi20479*, *Lchi00028*, *Lchi27918*, *Lchi30260*, *Lchi00012*, *Lchi22958*, *Lchi24398*, and *Lchi19924*. Blue rectangles represent exons, black lines represent introns, and red rectangles represent the positions where the event occurred.

In this study, we randomly selected 8 AS genes and used PCR to validate the AS events. We identified 12 AS events that occurred in these genes. Among them, two events occurred in *Lchi20479*, one event occurred in *Lchi00028*, three events occurred in *Lchi27918*, one event occurred in *Lchi30260*, one event occurred in *Lchi00012*, one event occurred in *Lchi22958*, two events occurred in *Lchi24398*, and one event occurred in *Lchi19924* (**Figure 11**). The AS events that were validated by PCR were consistent with our sequencing results, indicating that our analysis results based on hybrid sequencing data were reliable.

## Discussion

AS is an important post-transcriptional regulation mechanism that enables one gene to produce multiple splice variants, thus increasing the complexity of the transcriptome and proteome (Lee and Rio, 2015; Hartmann et al., 2016). Moreover, AS plays pivotal roles in plant growth and development, the stress response, flowering, biological rhythms, and signal transduction (Staiger and Brown, 2013; Wang et al., 2014; Sun and Xiao, 2015; Dong et al., 2018). In *A. thaliana*, a splice variant of *auxin response factor 8* has been found to play a role in regulating endothecium lignification and stamen elongation (Ghelli et al., 2018). *Plant intracellular Ras-group leucine-rich repeat protein 6* is necessary for female and male gametogenesis in *A. thaliana*, and it is regulated by AS (Forsthoefel et al., 2018). Moreover, many studies on AS have been performed in different plant species, such as *A. thaliana*, tomato, moso bamboo, and peanut (Wang et al., 2014; Wang et al., 2017; Ruan et al., 2018; Zhao et al., 2018; Clark et al., 2019). However, specific studies on AS in *L. chinense* remain scarce. In this study, we combined SMRT sequencing with Illumina sequencing to obtain transcriptome data from 21 *L. chinense* samples. After data processing and analysis, we obtained large amounts of information about AS in *L. chinense*. This is the first systematic study of AS in this species.

To our knowledge, a large proportion of genes undergo AS events in plants. In *A. thaliana*, more than 61% of multi-exon genes undergo AS events (Marquez et al., 2012). In maize, peanut, moso bamboo, and tomato, the proportions of AS genes are approximately 55%, 37%, 49%, and 65%, respectively (Min, 2017; Ruan et al., 2018; Zhao et al., 2018; Clark et al., 2019). Due to the limited sample size and sequencing depth in this study, we found that only 23.66% (8,293/35,055) of multi-exon genes in *L. chinense* were alternatively spliced (**Figure 1**). Thus, the proportion of alternatively spliced multi-exon genes in *L. chinense* is apparently lower than the proportions in the above species. Notably, however, the number of samples and the quality and depth of sequencing have been found to be positively correlated with the proportion of alternatively spliced multi-exon genes (Ruan et al., 2018). For example, with improvements in the quality and depth of sequencing, the proportion of AS genes has been found to increase from 22% to greater than 61% in *A. thaliana* (Reddy, 2007; Marquez et al., 2012). In moso bamboo, the AS rate has increased from ~36% to greater than 49%, mainly because of increases in sample size (Wang et al., 2018; Zhao et al., 2018). We believe that the proportion of known AS genes in *L. chinense* will increase with increasing sample size and sequencing depth.

Unlike in animals, the most common AS event in plants is IR rather than ES (Chaudhary et al., 2019b). In this study, IR (10,743, 28.39%) accounted for the highest proportion of AS events (**Figure 1F**). This finding is consistent with those obtained in studies on tomato, *A. thaliana*, and maize (Marquez et al., 2012; Min, 2017; Clark et al., 2019). Moreover, ES (6,312, 16.68%) was the third largest AS event (**Figure 1F**). Both ES and IR contribute to increasing the complexity of plant transcriptomes and proteomes. ES manifests its influence by affecting protein-protein interaction sites and post-translational modification sites (Buljan et al., 2012; Ellis et al., 2012). IR occurs in open reading frames (ORFs), affecting transcriptome and proteome complexity in two ways—one is by introducing PTCs into the ORF to trigger NMD or produce truncated proteins, and the other is by producing an elongated ORF that allows the production of pairs of protein isoforms (Jacob and Smith, 2017). Moreover, we identified 1,656 PTC^+^ transcripts and 3,310 PTC^-^ transcripts (**Figure 4A**). Actually, these 1,656 PTC^+^ transcripts may only partially trigger NMD, as a previous analysis showed that approximately one third of the PTC^+^ transcripts are likely to trigger NMD (Lewis et al., 2003). In addition, NMD effectors also play important roles in the degradation of PTC^+^ transcripts through the NMD pathway, including Upf proteins. Although we found that the expression patterns of the three Upf genes in pistils and leaves were opposite, we did not find any obvious relationship between Upf genes and PTC^+^ transcripts. However, we still believe that AS causes genes to produce multiple splice variants, substantially increasing the complexity and diversity of the *L. chinense* transcriptome and proteome.

Tissue-specific AS is controlled by SFs (Chen and Manley, 2009). SR proteins and hnRNPs are the main SFs that play roles in regulating AS (Vitulo et al., 2014). These two SFs play roles in different manners; SR proteins regulate AS by participating in spliceosome formation while hnRNPs usually act as repressors or activators (Mauger et al., 2008; Mai et al., 2016). Many SR proteins and hnRNPs have been reported to be involved in AS regulation, such as SRp38, SRp40, RBM39, hnRNP A1, hnRNP F, and hnRNP H, (Feng et al., 2009; Yan et al., 2010; Talukdar et al., 2011; Mai et al., 2016; Nazim et al., 2017). Differential expression of SR proteins and hnRNPs affects AS in different tissues (Chen and Manley, 2009; Vitulo et al., 2014). In grapes, differentially expressed SR proteins are related to tissue-specific AS (Vitulo et al., 2014). We found that 13 of the 14 differentially expressed SR and hnRNP genes showed opposite expression patterns in leaves and pistils, and their expression patterns were consistent with those of most AS genes (**Figure 3**). Furthermore, three Upf genes that play critical roles in the NMD pathway also showed an opposite expression pattern in these two tissues (**Figure 4D**). We speculate that differentially expressed SR and hnRNP genes and Upf genes may be related to the differences between pistils and leaves at the transcriptome level, further impacting the complexity of the transcriptome.

To further reveal the potential roles that AS may play in *L. chinense*, we performed WGCNA of the AS genes. As a powerful tool with which to reveal the relationships between different gene sets or between genes and phenotypes, WGCNA has been extensively applied in different fields, such as medicine and plant science (Wang J.-L. et al., 2019; Zhu et al., 2019; Esmaeili et al., 2020; Jia et al., 2020). We found that pistils and leaves may have huge differences in transcription and post-transcriptional regulation, because most blue-module genes that were related to transcription and post-transcriptional regulation were strongly expressed in pistils but weakly expressed in leaves (**Figures 6A**, **S3A**, **and** **S4A**). AS, as an important post-transcriptional regulation mechanism, also makes contributions to these differences, so AS-related SR genes, hnRNP genes, and Upf genes also presented different expression patterns in pistils and leaves. Moreover, AS genes from different coexpression modules participated in multiple physiological and biochemical processes, such as fatty acid biosynthesis and metabolism, galactose metabolism, and photosynthesis, and these processes were necessary for plant life. Furthermore, 15 AS TFs were identified as hub genes in four modules. Therefore, the effects of AS on *L. chinense* were varied, and these findings have broadened our view on AS of *L. chinense*.

To identify the differences between AS genes and non-AS genes, we compared these gene types and found that AS genes had more exons and introns and produced longer transcripts than did non-AS genes (**Figures 10A, B**). The number of exons/introns was positively related to the number of AS events (**Figures 9E, F**). These results indicate that genes with more complex structures tend to experience more AS events, which is consistent with the findings obtained in study on moso bamboo (Wang et al., 2018). However, there were no significant differences in splicing sites between AS genes and non-AS genes (**Figures 8E, F**). Similarly, in tomato, moso bamboo, and *A. comosus* var. *bracteatus*, researchers have reported that AS genes and non-AS genes have no differences in splicing site selection (Zhao et al., 2018; Clark et al., 2019; Ma et al., 2019). Moreover, we found that short exons tended to be skipped, while short introns tended to be retained (**Figures 10A, B**). The same phenomenon has been observed in tomatoes (Clark et al., 2019). We believe that the structural features of genes may affect AS. These findings greatly increase our understanding of AS in *L. chinense*.

AS can not only increase the complexity of the transcriptome and proteome but also influence lncRNAs (Kiegle et al., 2018; Yang et al., 2019). LncRNAs are critical regulators of plant growth and development, flowering, the stress response, and other biological processes (Liu et al., 2018; Ayachit et al., 2019). In grapevine, researchers have identified 813 differentially expressed lncRNAs related to the cold stress response (Wang P. et al., 2019). Moreover, researchers have found that more than 72% of lncRNAs in tomatoes undergo AS events (Yang et al., 2019). In our study, we identified 183 lncRNAs associated with AS (**Figure 10D**). We believe that the different sequencing and analysis methods used in our study may have restricted our detection of alternatively spliced lncRNAs in *L. chinense*. However, our findings still expand the current understanding of AS in *L. chinense*.

## Conclusion

In this study, we used different tools and algorithms to effectively obtain information about AS in *L. chinense* from hybrid sequencing data. We identified a total of 48,408 genes, 8,503 of which were found to undergo AS. IR events (10,743, 28.39%) were the most common event, resulting in the production of 1,656 PTC^+^ and 3,310 PTC^-^ transcripts. Furthermore, we detected 183 lncRNAs that were alternatively spliced. These findings indicate that AS plays a critical role in increasing the complexity of the transcriptome of *L. chinense.* Moreover, WGCNA revealed that pistils and leaves had great differences in the activities of transcription and post-transcriptional regulation, and AS had an impact on many physiological and biochemical processes in *L. chinense*. We also found that structural features of genes may affect AS. AS genes and non-AS genes had differences in transcript length, exon number, and exon/intron length, and AS genes with more exons or introns experienced more AS events. Furthermore, shorter introns tended to be retained, while shorter exons tended to be skipped. To validate our results, we randomly selected 8 AS genes for PCR, and the PCR results showed that our analysis was reliable. Our study provided new information about AS in *L. chinense*, and it is the first to comprehensively report on AS in this species. Our data thus provide a reference for further study of AS in *L. chinense*.

## Data Availability Statement

The datasets presented in this study can be found in online repositories. The names of the repository/repositories and accession number(s) can be found below: https://www.ncbi.nlm.nih.gov/, PRJNA559687.

## Author Contributions

Experimental design: ZT and HL. Plant material collection and execution of the experiments: ZT, YS, SW, and YZ. Data analysis: ZT, YS, and SW. Manuscript writing: ZT and YZ. All authors contributed to the article and approved the submitted version.

## Funding

The design of the study, sample collection, hybrid sequencing, and data analysis and interpretation were supported by the National Natural Science Foundation of China (31770718, 31470660) and the Priority Academic Program Development of Jiangsu Higher Education Institutions (PAPD).

## Conflict of Interest

The authors declare that the research was conducted in the absence of any commercial or financial relationships that could be construed as a potential conflict of interest.
