# Supplementary figures and images for: Alternative Splicing Enhances the Transcriptome Complexity of Liriodendron chinense
Source: Front Plant Sci. 2020 Sep 23;11:578100. doi: 10.3389/fpls.2020.578100 (PMC7539066; doi:10.3389/fpls.2020.578100)

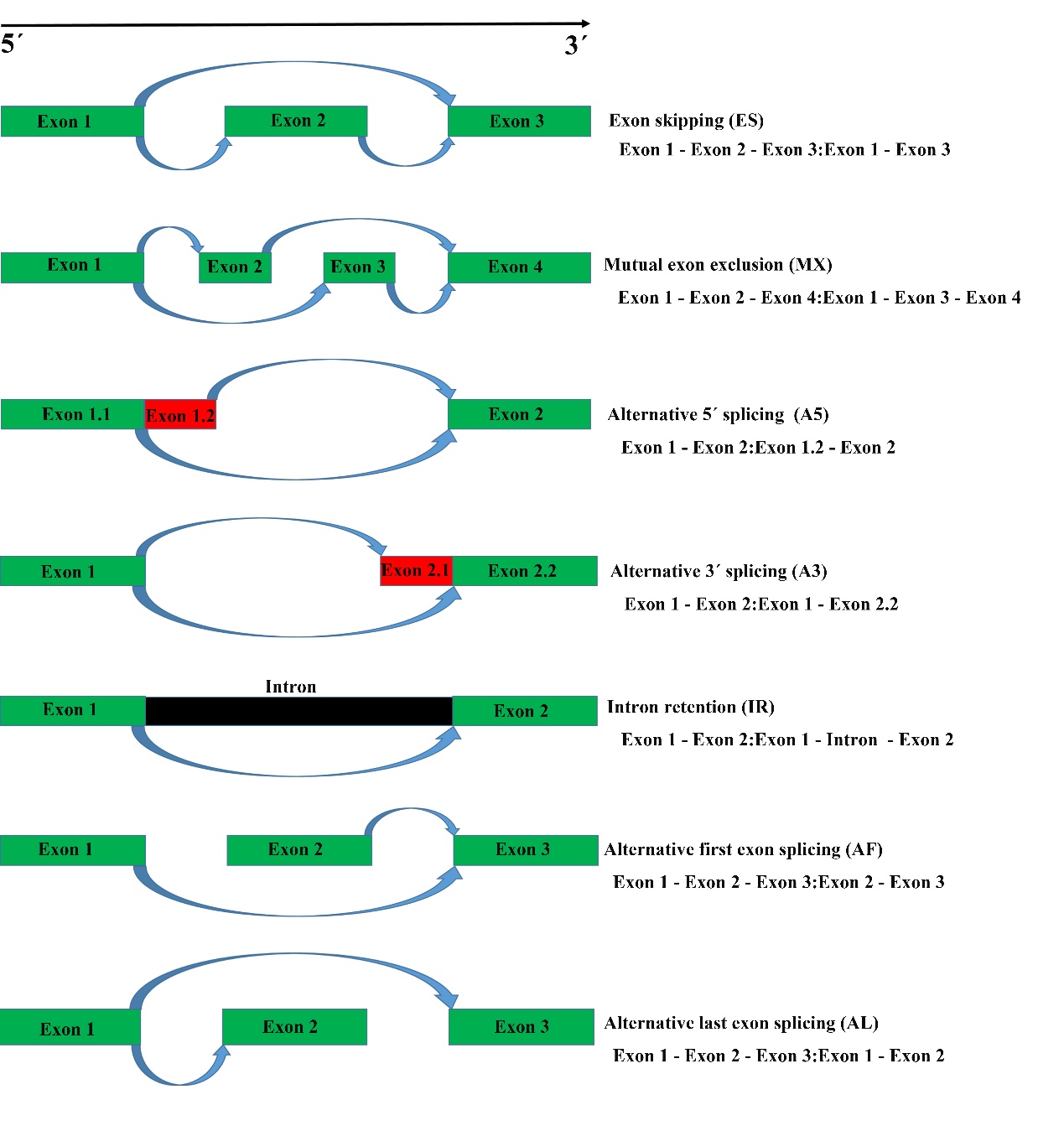


**Figure S1.** AS events classification by SUPPA software.

Supplement: Supplementary file 1 [file Table_1.doc]
