# Supplementary material for: Alternative Splicing Enhances the Transcriptome Complexity of Liriodendron chinense
Source: Front Plant Sci. 2020 Sep 23;11:578100. doi: 10.3389/fpls.2020.578100 (PMC7539066; doi:10.3389/fpls.2020.578100)
Supplement: Supplementary file 2 [file Table_2.doc]

**Table S1** Primers for PCR

| Gene | Forward primer (5′- 3′) | Reverse primer (5′- 3′) | Tm (℃) |
| --- | --- | --- | --- |
| Lchi20479 | AAAAGGTTTCAATCGACTGTGG | TGGCAATGGATAACATCTTCGG | 61.5 |
| Lchi00028 | AAAGCCTACTCGCACTCTTCGT | TCTCTCCTGGGCACAAGCAC | 62.0 |
| Lchi27918 | CCGGCCCTCGAAATGACTCTC | CACACCTTGCTCATGTACCCAC | 62.5 |
| Lchi30260 | TCATTCTCGACAAATCATCAGGG | AACCATTATTAGAAAACGGCAGA | 57.5 |
| Lchi00012 | AGGAGGTTTATCAAGTAATCTGGG | GCTTTTTCAGGCCATCTCTTCA | 58.5 |
| Lchi22958 | CGCAAGATTTCCACCCGAAGCA | CACGTTCGCCCATCATAACCC | 62.0 |
| Lchi24398 | TCCGTAAAACTTCACACTAACCC | TCAACACTGTCGTTACGCTTC | 58.5 |
| Lchi19924 | TGCGCTCTGATACTGAACACC | CCTTCCTCTTTTATCGCATCCAC | 59.0 |
