# Supplementary material for: Alternative Splicing Enhances the Transcriptome Complexity of Liriodendron chinense
Source: Front Plant Sci. 2020 Sep 23;11:578100. doi: 10.3389/fpls.2020.578100 (PMC7539066; doi:10.3389/fpls.2020.578100)
Supplement: Supplementary file 3 [file Table_3.doc]

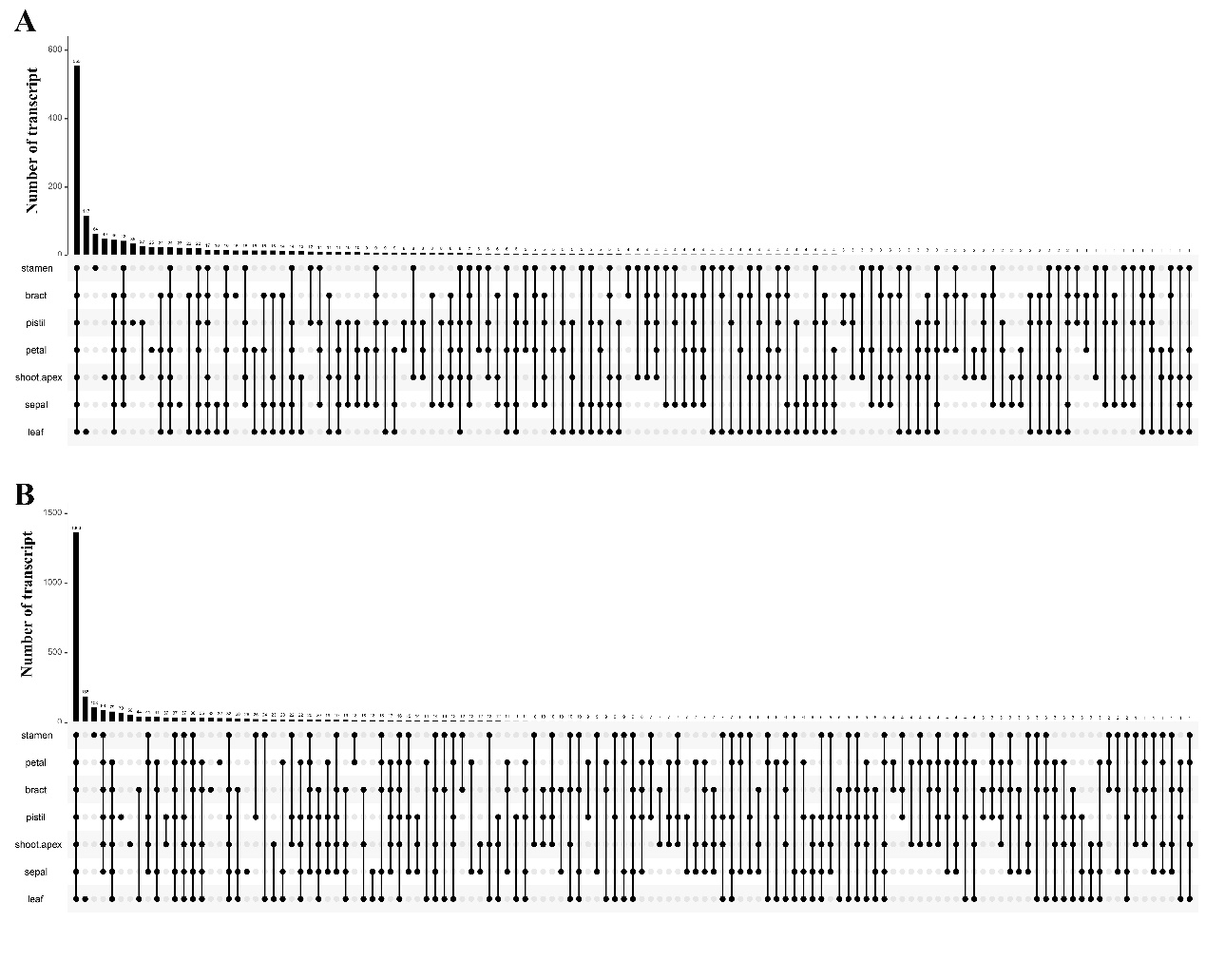


Figure S2. UpSet plot of PTC-containing and non-PTC-containing transcripts. (A) UpSet plot of PTC-containing transcripts. (B) UpSet plot of non-PTC-containing transcripts.
