# Supplementary material for: Alternative Splicing Enhances the Transcriptome Complexity of Liriodendron chinense
Source: Front Plant Sci. 2020 Sep 23;11:578100. doi: 10.3389/fpls.2020.578100 (PMC7539066; doi:10.3389/fpls.2020.578100)
Supplement: Supplementary file 5 [file Table_5.doc]

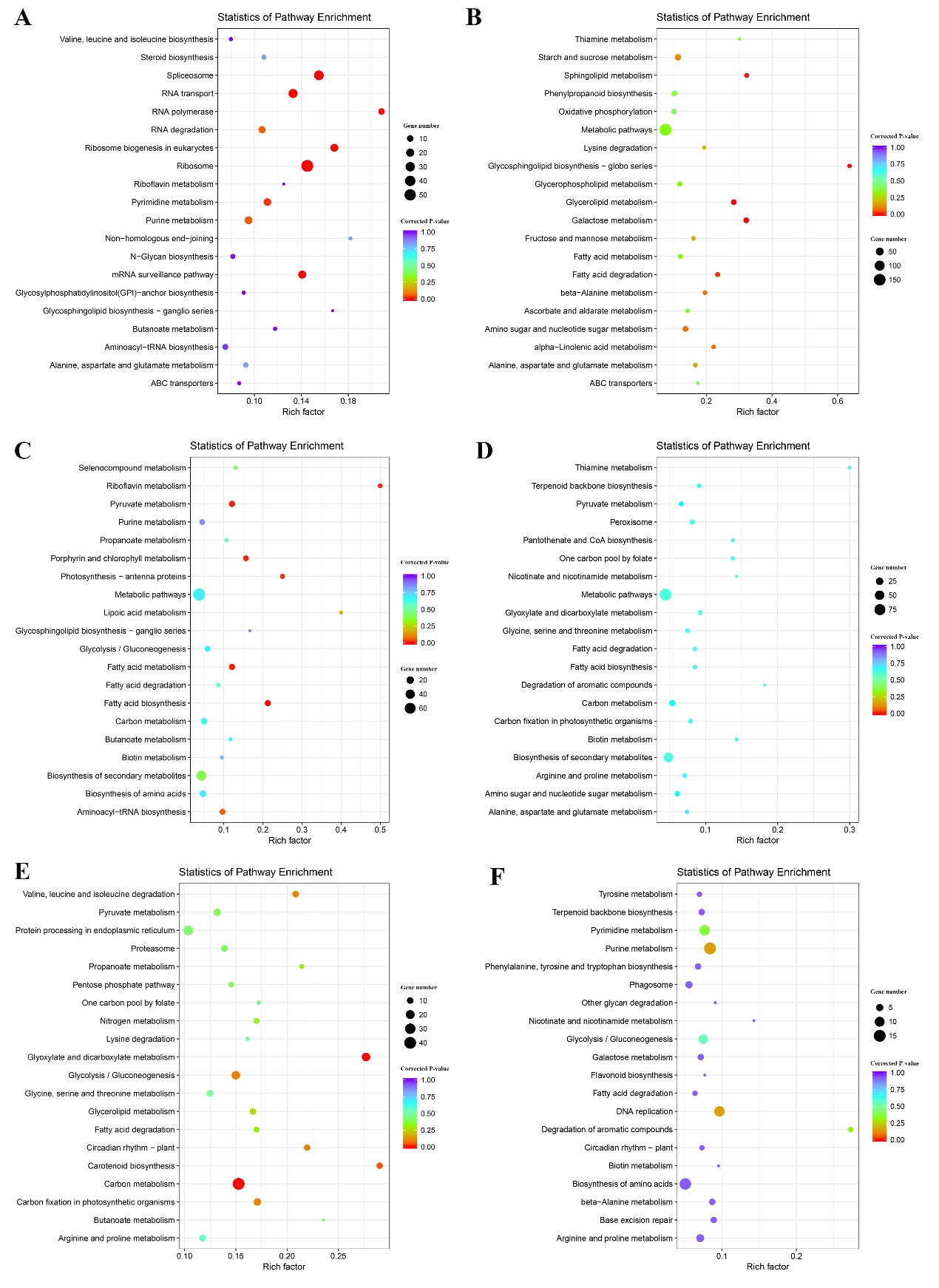


**Figure S4** KEGG pathway enrichment analysis of co-expressed AS genes from six modules. Only the top 30 terms are shown according to the ranking of corrected P-values. **(A)**-**(F)** KEGG pathway enrichment analysis of AS genes from the blue, brown, green, grey, turquoise, and yellow modules, respectively.
